# Kimura’s disease of the lacrimal gland mimicking IgG4-related orbital disease

**DOI:** 10.1186/1471-2415-14-158

**Published:** 2014-12-15

**Authors:** Jing Li, Xin Ge, Jianmin Ma, Ming Li, Jinru Li

**Affiliations:** Beijing Tongren Eye Center, Beijing Tongren Hospital, Capital Medical University, Beijing Ophthalmology and Visual Sciences Key Laboratory, No. 1 Dongjiaominxiang Street, Dongcheng District, Beijing, China 100730; Beijing Institute of Ophthalmology, Beijing Tongren Hospital, Capital Medical University, Beijing, China; Department of Pathology, Beijing Tongren Hospital, Capital Medical University, Beijing, China

**Keywords:** Kimura’s disease, IgG4-related disease, Lacrimal gland

## Abstract

**Background:**

Kimura’s disease (KD) is a rare and benign chronic inflammatory soft tissue disorder of unknown origin, which predominantly inflicts young male adults in Asia. IgG4-related disease is a new disease concept, established this century and characterized by fibrosis and sclerosis of the involved organs, with infiltration of IgG4-positive plasma cells. These two kinds of diseases share similar characteristics, which may complicate their diagnosis.

**Case presentation:**

A 47-year-old Chinese man presented to our Department of Ophthalmology with a 26-month history of painless swelling and redness left upper eyelid. Surgical excisions of the left lacrimal gland were performed. A histopathology examination showed follicular hyperplasia with reactive germinal centres and eosinophilic infiltration involving the interfollicular areas as well as proliferation of post capillary venules, all signs of Kimura disease. Immunohistochemical analysis of the cells demonstrated positive staining for CK, Vimentin, CD3, CD4, CD20, CD21, CD117, CD5, CD8, CD23, IgG and IgG4 (30 per high-power field) and negative staining for CD10 and CD34. Some ophthalmologists in our department questioned whether the histological and immunohistochemical findings were also compatible with features of IgG4-related diseases. There was no sign of recurrence during the twelve months of regular follow-up.

**Conclusion:**

Kimura’s disease may present with high serum IgG4 levels, which may be an epiphenomenon related to chronic antigen exposure. As clinical doctors, especially ophthalmologists, we should recognize the possibility of the occurrence of increased serum levels of IgG4 in Kimura’s disease to ensure correct diagnosis.

## Background

Kimura’s disease (KD) is a rare and benign chronic inflammatory soft tissue disorder of unknown origin, which predominantly inflicts head and neck of young male adults in Asia [1, 2]. IgG4-related disease is a new disease concept, established this century and characterized by fibrosis and sclerosis of the involved organs, with infiltration of IgG4-positive plasma cells [3, 4]. Herein, we report a case of KD occurring in the left lacrimal gland with increased serum levels of IgG4, which mimics IgG4-related disease. To our knowledge, few publications in the literature have been reported with KD of lacrimal gland likewise.

## Case presentation

A 47-year-old Chinese man presented to our department of ophthalmology with a 26-month history of left upper eyelid painless swelling and redness. The patient was initially treated with a course of intravenous penicillin, which resulted in temporary regression of the lesion. There had been no similar family history of this appearance to date.

**Figure 1:**
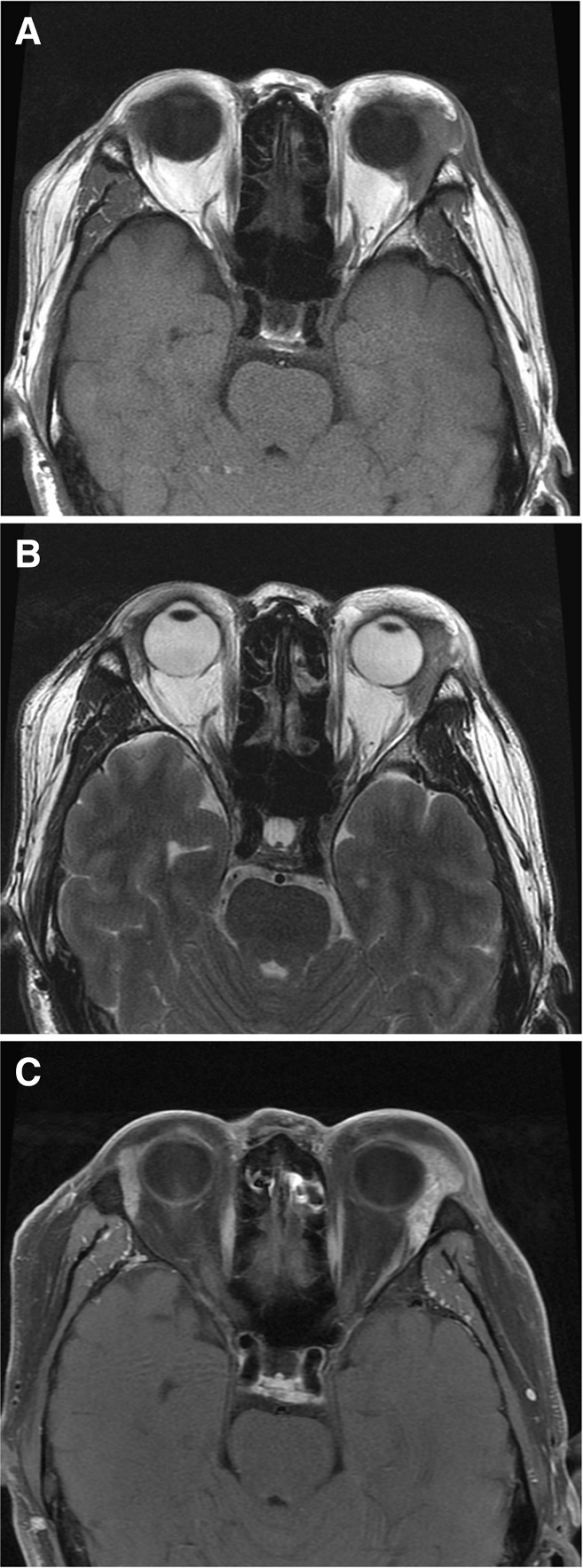
**Magnetic resonance imaging (MRI) examination of the orbit showed that the left lacrimal gland was lobulated lobulated with a distinct margin and was predominantly isointense on T1-weighted images (A), isointense on T2-weighted images (B) and an obvious heterogeneous enhancement on contrast-enhanced MRI images (C).**

**Figure 2:**
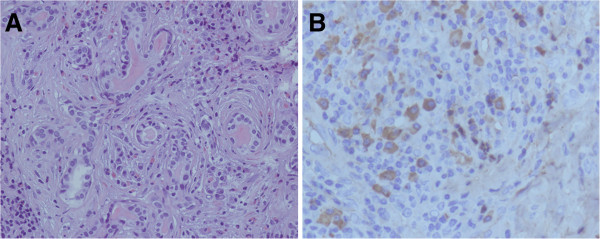
**Histopathological examinations of the patient’s left lacrimal gland lesion. A**: Hematoxylin-eosin staining demonstrated follicular hyperplasia with evidence of reactive germinal centres. Eosinophilic infiltration, involving the interfollicular areas, and proliferation of post capillary venules were observed; ×200. **B**: Immunohistochemically, the cells showed positive for IgG4 (30 per high-power field); ×400.

Physical examination revealed that the left lacrimal gland was obviously swelling, which presented with a soft, non-tender and smooth-surfaced. Slit lamp examination was otherwise unremarkable aside from bilateral mild cataract. Laboratory testing revealed a white blood cell count of 9.8 × 10^-9^/L with 15.91% eosinophils(normal range: 0.05-0.5 × 10^-9^/L). Serum concentration of immunoglobulin E was elevated at 469.3 IU/ml, and immunoglobulin G4 was 295 mg/dL (normal range less than 135 mg/dL). Magnetic resonance imaging (MRI) examination of the orbit showed that the left lacrimal gland was lobulated with a distinct margin and was predominantly isointense on T1-weighted images. Isointense on T2-weighted images and an obvious heterogeneous enhancement on contrast-enhanced MRI images are shown in Figure 1. The chest radiograph was normal and no cervical lymph nodes were palpable.Next, we advised the patient to be hospitalized and the patient underwent an orbital biopsy. Intraoperative frozen sections were reported as being consistent with a benign tumor. Therefore, a complete resection was undertaken. Perioperatively, it was noted that the mass had a smooth and homogeneous grey appearance. There was no evidence of haemorrhage or cystic change and borders were indistinct. A definite diagnosis of KD was made by histopathology and immunohistochemical examinations of representative resected specimens after surgery. Histopathological examination demonstrated follicular hyperplasia with evidence of reactive germinal centres. Eosinophilic infiltration, involving the interfollicular areas, and proliferation of post capillary venules were observed. These features enabled a diagnosis of KD to be made (Figure 2A). Further analysis, including immuohistochemistry, was undertaken, demonstrating positive staining for CK, Vimentin, CD3, CD4, CD20, CD21, CD117, CD5, CD8, CD23, IgG and IgG4 (30 per high-power field) and negative staining for CD10 and CD34 (Figure 2B), the ratio of IgG4 to IgG positive cells was about 35%. The pathological diagnosis, which was also the final diagnosis, was KD. However, some ophthalmologists questioned whether the histological and immunohistochemical findings were also had common features with IgG4-related disease, which is a newly recognized fibro-inflammatory condition characterized by tumefactive lesions, a dense lymphoplasmacytic infiltrate rich in IgG4-positive plasma cells, storiform fibrosis, and elevated serum IgG4 concentrations.

The patient was discharged following his surgery and treated with prednisone 25 mg/day for four weeks and then reduce by 5 mg/day every two weeks. His symptoms gradually improved. Serum immunoglobulin E (IgE) levels dropped to 125.9 IU/mL and eosinophil’s decreased to 0.69 × 10^9^/L after 2 months. There was no sign of recurrence in twelve months of regular follow-up.

## Discussion

Kimura disease (KD) is a rare benign chronic inflammatory disease of unknown etiology, which frequently affects young male adults in Asia. It often presents with subcutaneous masses around the head and neck without systemic symptoms. Laboratory tests are characterized by eosinophilia (>5% of white blood cell, or >0.5 × 10^9^/L of blood) and raised IgE levels [1, 2].

Immunoglobulin G4-related disease (IgG4-RD) is a recently defined systemic fibro inflammatory condition characterized by elevated serum levels of IgG4 and abundant infiltration of IgG4-bearing plasma cells and fibrosis of the involved organs. It can inflict all the organs, such as pancreas, bile duct, gallbladder, orbit, salivary gland, retro peritoneum, and kidney [3, 4].

Diagnosis of any diseases should be carried out in a comprehensive manner on the basis of physical, imaging, serological and, especially, histopathological findings. However, there are some similar characteristics between KD and IgG4-RD, with eosinophilia or high IgE levels often found in patients with IgG4-RD [5–8]. Herein, we have reported a case study of a 47-year-old man with a 26-month history of left upper eyelid painless swelling and redness. He had a significant eosinophilia and elevated serum IgE and IgG4 levels. Biopsy of his left lacrimal gland suggested the diagnosis of both KD and IgG4-RD. Therefore, we performed a literature review of Kimura’s disease, in which elevated serum levels of IgG4 and/or abundant infiltration of IgG4-bearing plasma cells had also been reported. To our knowledge, there were 5 cases that reported Kimura’s disease with the characteristics of IgG4-RD, which were located in skin, lung, and lymph node [5–8]. No orbital KD has been reported to date.

Although the two kinds of diseases share some similarities, there are still several different features. Expression of IgE in follicular dendritic cells and eosinophilic micro abscesses is routinely seen in Kimura’s disease, but not in IgG4-related disease [8]. High serum IgG4 concentration and marked infiltration of IgG4-positive plasma cells is also seen in IgG4-related disease, but not in KD. Tsubouchi and colleagues observed numerous IgG4-positive plasma cells in a lung biopsy of a patient with Kimura’s disease, and they suggested the coexistence of the two diseases [6]. Even using the low threshold of 10 IgG4 + cells/high powered field, IgG4 staining correlates with marked eosinophilia in the lacrimal gland based on histology and gene expression. Lei Liu and colleagues reported a 23-year-old Chinese man with remarkable eosinophilia and elevated serum IgE levels [7]. Biopsy of his lymph nodes suggested the diagnosis of both Kimura’s disease and IgG4-related disease. They analyzed the relationship between the two diseases and held a view different from Tsubouchi, that the presence of any IgG4-positive plasma cells in the peripheral blood, infiltration of IgG4-positive plasma cells in lymph nodes, and skin lesions are epiphenomena of Kimura’s disease. We agree with the opinion of Lei Liu and his colleagues.

Recently, Wong et al. analyzed gene expression and the prevalence of IgG4-immunostaining among subjects with orbital inflammatory diseases and made a conclusion that IgG4 + plasma cells are common in orbital tissue from patients with sarcoidosis, granulomatosis with polyangiitis (GPA), or nonspecific orbital inflammatory disease (NSOI) [9]. Aithough KD patients was not collected in his study, the investigation was a indirect support to our opinion.

In summary, we report a rare case of Kimura’s disease occurring in the left lacrimal gland with increased serum levels of IgG4 mimicking IgG4-related disease. Although rare, as clinical doctors, especially ophthalmologists, we should recognize the possibility of occurrence of KD with increased serum levels of IgG4 and pay attention to the disease in our clinical work. Only in this manner can we diagnose it correctly when we meet this situation and then choose the best treatment strategy in order to decrease the rate of recurrence.

## Conclusion

Kimura’s disease may present with high serum IgG4 levels, which is just an epiphenomenon related to chronic antigen exposure. As clinical doctors, especially ophthalmologists, we should recognize the possibility of the occurrence of increased serum levels of IgG4 in Kimura’s disease and diagnose the disease correctly.

### Consent

Written informed consent was obtained from the patient for publication of this Case report and any accompanying images. A copy of the written consent is available for review by the Editor of this journal.
